# White Matter Integrity and Motor Function Disruption Due to Traumatic Brain Injury in Piglets: Impacts on Motor-Related Brain Fibers

**DOI:** 10.3390/brainsci14030247

**Published:** 2024-03-02

**Authors:** Madison M. Fagan, Kelly M. Scheulin, Sydney E. Sneed, Wenwu Sun, Christina B. Welch, Savannah R. Cheek, Erin E. Kaiser, Qun Zhao, Kylee J. Duberstein, Franklin D. West

**Affiliations:** 1 Regenerative Bioscience Center, University of Georgia, 425 River Rd., Athens, GA 30602, USA; madison.fagan25@gmail.com (M.M.F.); kelly.scheulin@gmail.com (K.M.S.); sydney.sneed@uga.edu (S.E.S.); ws40404@uga.edu (W.S.); erin1019@uga.edu (E.E.K.); qunzhao@uga.edu (Q.Z.); kyleejo@uga.edu (K.J.D.); 2 Neuroscience Program, Biomedical and Health Sciences Institute, University of Georgia, 500 D.W. Brooks Dr., Athens, GA 30602, USA; 3 Department of Animal and Dairy Science, College of Agriculture and Environmental Science, University of Georgia, 425 River Rd., Athens, GA 30602, USA; christina.welch@uga.edu (C.B.W.); savannah.cheek@uga.edu (S.R.C.); 4 Department of Physics and Astronomy, Franklin College of Arts and Sciences, Astronomy Building 1003, University of Georgia, Sanford Dr., Athens, GA 30602, USA

**Keywords:** pediatric TBI, diffusion tensor imaging, motor function, piglet model

## Abstract

Pediatric traumatic brain injury (TBI) often induces significant disability in patients, including long-term motor deficits. Early detection of injury severity is key in determining a prognosis and creating appropriate intervention and rehabilitation plans. However, conventional magnetic resonance imaging (MRI) scans, such as T2 Weighted (T2W) sequences, do not reliably assess the extent of microstructural white matter injury. Diffusion tensor imaging (DTI) tractography enables three-dimensional reconstruction of specific white matter tracts throughout the brain in order to detect white matter injury based on anisotropic diffusion. The objective of this study was to employ DTI tractography to detect acute changes to white matter integrity within the intersecting fibers of key motor-related brain regions following TBI. Piglets were assigned to either the sham craniectomy group (sham; *n* = 6) or the controlled cortical impact TBI group (TBI; *n* = 6). Gait and MRI were collected at seven days post-surgery (DPS). T2W sequences confirmed a localized injury predominately in the ipsilateral hemisphere in TBI animals. TBI animals, relative to sham animals, showed an increased apparent diffusion coefficient (ADC) and decreased fractional anisotropy (FA) in fiber bundles associated with key brain regions involved in motor function. TBI animals exhibited gait deficits, including stride and step length, compared to sham animals. Together these data demonstrate acute reductions in the white matter integrity, measured by DTI tractography, of fibers intersecting key brain regions that strongly corresponded with acute motor deficits in a pediatric piglet TBI model. These results provide the foundation for the further development of DTI-based biomarkers to evaluate motor outcomes following TBI.

## 1. Introduction

Pediatric traumatic brain injury (TBI) is one of the most common neurological injuries in children. Recently, the Centers for Disease Control and Prevention reported TBI resulted in 640,000 emergency department visits, 18,000 hospital stays, and 1500 deaths per year among children under 14 years old [1]. The pediatric population is uniquely affected by TBI as it often interrupts and irrevocably alters ongoing neural development, leading to long-term motor deficits, such as reduced balance and coordination [2,3,4]. Furthermore, decreased white matter integrity in specific structures (i.e., the pyramidal tract) is strongly correlated with poor gait and motor performance following pediatric TBI [4,5,6,7,8]. Therefore, early detection and evaluation of affected white matter within specific brain regions is critical in developing appropriate interventional plans that lead to improved long-term outcomes [9].

Magnetic resonance imaging (MRI) is a commonly used neuroimaging technique that reliably identifies cerebral lesioning, edema, hemorrhage, and other pathophysiological changes resulting from TBI [10]. In translational animal models, recent research has shown that increased lesion volumes are highly correlated with reduced step length, stride length, and poor outcomes following TBI [11,12]. Moreover, in a pig model of brain injury, motor-related brain structures most impacted by a stroke were shown to be predictive of functional deficits, as measured by gait and behavior assessments [13]. Though conventional MRI scans (e.g., T2 Weighted) are commonly used neuroimaging tools following injury, this approach lacks the sensitivity needed to reliably detect microstructural white matter injury [14]. This injury mechanism is critically important to consider as axonal injury is a common cause of motor deficits following TBI [5,7]. Thus, more sensitive assessments of white matter damage are essential to better predict TBI outcome, especially regarding motor function.

Diffusion tensor imaging (DTI) enables specific assessment of microstructural white matter integrity by measuring the movement of water molecules in the brain [15]. Furthermore, DTI tractography allows for three-dimensional (3D) reconstruction and characterization of microstructural injury to specific fiber tracts [16,17,18]. This technique collects data based on the premise that healthy, intact axons limit the free movement of water molecules to primarily travel parallel to the axon, in part due to the high degrees of myelination of white matter. Therefore, the anisotropy, or directionality of diffusion, of white matter fiber tracts can be investigated throughout the brain following TBI to evaluate the presence of axonal injury that is undetectable utilizing conventional MRI sequences [19]. DTI has been implicated as a particularly valuable tool in assessing white matter damage following pediatric TBI as it is sensitive to developmental changes (i.e., the level of axon myelination for a given age) [9,20]. Fractional anisotropy (FA) is a common DTI measure that quantifies the degree of anisotropy of water diffusion, while apparent diffusion coefficient (ADC) measures the overall magnitude of water diffusion. Lower FA and higher ADC values are associated with loss of white matter integrity [21]. These measures have been shown to be reliable indicators of microstructural changes to fiber tracts following TBI.

DTI tractography with regional specificity has shown efficacy in detecting microstructural white matter fiber tract damage. Multiple clinical studies have demonstrated interruptions to the pyramidal motor white matter tract (consisting of fibers from the primary motor cortex, premotor cortex, somatosensory areas, and basal ganglia, among others) and white matter in other motor-related brain structures in pediatric and adult patients who presented motor weakness or gait disturbances after TBI [5,6,7,8,22,23,24]. Ressel et al. were the first to report that mean FA of the ipsilateral pyramidal tract demonstrated proficiency in predicting motor scores of Functional Independence Measurement in children following TBI [25]. A recent preclinical TBI rodent study determined subacute decreased FA in the perilesional cortex and ipsilateral hippocampus may be a promising predictive biomarker for chronic functional deficits, including impaired learning and a reduction in distance moved as measured by Morris Water Maze and open field performance, respectively [26]. However, to date, no group has performed comprehensive, regionally specific DTI tractography analysis to determine the impact of a unilateral TBI on ADC and FA as well as the subsequent effect on gait deficits in a pediatric piglet TBI model.

Due to key parallels with humans in brain anatomy and physiology, the porcine TBI model is a robust and predictive animal model [27]. Pig and human brains possess a high level of gyrification as opposed to the lissencephalic rodent brain, thus resulting in greater heterogeneity of TBI. The presence of sulci transfers the external force away from the cortex and into the base of the sulci as opposed to an even force distribution as observed in lissencephalic brains [28,29,30]. Pig and human brains are comprised of >60% white matter, while commonly used rodent brains are comprised of <12% white matter [31,32]. Reduced vascularization and increased anisotropy in white matter often leaves it more susceptible to injury as compared to grey matter; therefore, pigs are likely to develop white matter injuries that are more similar to humans [27]. Brain connectivity between humans and pigs is also comparable, thus allowing for a more direct comparison of functional neural networks [33,34,35]. Furthermore, the piglet brain shows a more similar progression of neural development, maturation, and myelination to human adolescents compared to rodent models; consequently, it allows for an age-dependent response to injury that is more appropriate for modeling pediatric TBI [36,37]. Our research team has recently demonstrated that the piglet TBI model shows gait deficits comparable to pediatric TBI patients, including loss of coordination and reduced motor control [11,38,39,40]. These key similarities in human and porcine neural and functional responses to TBI make the pig a valuable tool in assessing novel neuroimaging sensitivity to white matter integrity post-TBI.

The purpose of this study was to employ DTI tractography to detect acute changes in the white matter integrity of key motor-related regions of interest and to determine functional deficits in a pediatric piglet TBI model. For the first time, this study provides evidence that DTI tractography is a sensitive tool that can detect microstructural changes in the white matter integrity of specific motor-related structures following moderate–severe TBI in a piglet model. Furthermore, this study establishes a possible connection between the changes in white matter integrity, as measured by ADC and FA values, of specific motor-related structures and gait deficits in a piglet TBI model. These findings suggest DTI analysis may be an advantageous tool in detecting the loss of white matter integrity in specific motor-related structures following a pediatric TBI.

## 2. Materials and Methods

### 2.1. Animals and Housing

Four-week-old male castrated Yorkshire crossbreed pigs (*n* = 12) were used as experimental animals in this study. TBI was induced in six (TBI; *n* = 6) piglets, while the remaining six underwent sham craniectomy surgery (sham; *n* = 6). The required sample size was based on our previous study data and was calculated by paired *t*-tests using two-tailed tests, α = 0.05, and a desired 80% power of detection with an effect size of 1.15. Piglets were housed in Public Health Service (PHS) and Association for Accreditation of Laboratory Animal Care (AAALAC) approved facilities maintained at room temperature (27 °C) with a 12 h light/dark cycle. Pigs were provided ad lib water access and fed standard pig starter I diets. Furthermore, pigs received daily enrichment through human contact and toys.

This study was performed in accordance with the National Institutes of Health (NIH) Guide for the Care and Use of Laboratory Animals. All procedures were reviewed and approved by the University of Georgia Institutional Animal Care and Use Committee (Animal Use Protocol: A2019-07-007-A25).

### 2.2. Controlled Cortical Impact

A moderate–severe controlled cortical impact (CCI) was administered as previously described [38,39,41]. Briefly, pigs were initially anesthetized with an intramuscular injection of xylazine (0.2 mg/kg, AnaSed, VETone, Boise, ID, USA) and midazolam (2 mg/kg, Covetrus NA, Portland, ME, USA), followed by an intravenous administration of propofol (0.5 mg/kg to effect, PropoFlo, Zoetis, Parsnippany, NJ, USA). Once corneal and noxious reflex was absent, pigs were intubated and maintained on 2–4% inhalant isoflurane (Abbot Laboratories, Chicago, IL, USA) in oxygen. Ophthalmic ointment (Dechra, Overland Park, KS, USA) was applied to avoid drying of or damage to the eyes. Body temperature was monitored via rectal thermometer and maintained via forced air heating system; heart rate and respiration were monitored routinely by trained personnel before, during, and after surgery until pigs were ambulatory. After sterile preparation of the skin overlaying the cranium, a 4 cm skin incision was made to expose the skull. A periosteal block (2 mg/kg, Nocita, Elanco, Greenfield, IN, USA) was applied and an approximately 20 mm diameter craniectomy was performed 3 mm rostral to the left anterior junction of the coronal and sagittal sutures. Pigs were then secured in a CCI device and a moderate–severe TBI was induced over the left motor cortex with a 15 mm diameter blunt impactor tip (velocity: 4 m/s, depth: 9 mm, dwell time: 400 ms). Sham pigs received a craniectomy only. The TBI site was flushed well with normal saline and the skin was reopposed with polyglactin 910 suture (Coated Vicryl, ETHICON, Raritan, NJ, USA). After surgery, all pigs were monitored until vitals returned to normal, every 4 h for 24 h, and twice daily for the remainder of the study.

### 2.3. Magnetic Resonance Imaging

All pigs (*n* = 12) underwent MRI at 7 days post-surgery (DPS) using a GE 32-channel fixed-site Discovery MR750 3.0 Tesla magnet and 8-channel knee coil. The following three sequences were acquired using the respective parameters:

- 3D Fast SPoiled GRadient echo (FSPGR) T1-Weighted (T1W) (FA = 9°, number of echoes = 1, TI = 900 ms, receiver bandwidth = 31.25 kHz, FOV = 12.8 × 12.8 × 6.4 cm, slice thickness = 1 mm, and a matrix size of 256 × 256 × 112);
- Fast Spin Echo (FSE) T2W (TR = 5.3 s, TE = 124 ms, echo train length = 17, receiver bandwidth = 20.83 kHz, FOV = 12.8 × 12.8 cm, matrix size of 384 × 224, slice thickness = 3 mm);
- Spin Echo (SE) DTI EPI (TR = 10.0 s, TE = min-full, FOV = 12.8 × 12.8 × 6.4 cm, a matrix size of 64 × 64 × 32, 3 b = 0 images, 30 diffusion weighted images, b = 1000 s/mm^2^).

All pigs were sedated using the same protocol as craniectomy surgery and maintained with mild anesthesia using isoflurane in oxygen (1.5%, Abbot Laboratories) for the duration of the scan.

### 2.4. Data Processing

Lesion volume was calculated from coronal T2W sequences using Osirix Software (Version 12.5.2). Hypointense and hyperintense areas in ipsilateral tissue slices were manually traced. Computer generated lesion volumes were then calculated and reported for sham and TBI animals.

Digital Imaging and Communications in Medicine (DICOM) images of anatomical T1W and DTI series were converted into Neuroimaging Informatics Technology Initiative (NIfTI) format using the “dcm2niix” software MRIcroGL version 20 July 2022 (v1.2.20220720) [42]. Brain masks were manually drawn slice-by-slice in 3D Slicer version 4 (slicer.org) [43].

One pig from the sham group was selected as the template pig based on visual inspections of the DTI and T1W data, taking into consideration distortion and artifacts. Each remaining pig’s DTI volumes were spatially normalized to the template pig using the first volumes of each pig that had no diffusion gradients (b = 0) applied. Spatial normalization was accomplished using Statistical Parametric Mapping’s (SPM) Old Normalize algorithm (SPM12, Institute of Neurology, University College London) [44]. The calculated transformation was applied to the rest of the prior motion-corrected volumes and the filed information of that corresponding pig. The superset was created by concatenating all of the pig’s corresponding DTI volumes at each voxel.

A porcine brain atlas [45] was then spatially normalized to each T1W, which was aligned with the DTI images. First, a spatial transformation was calculated between the pig brain atlas’ associated T1W anatomical image and each pig’s masked T1W image using the Old Normalize SPM algorithm. Then the calculated spatial transformation was applied to the atlas. Spatial transformations consisted of a 12-parameter affine transformation followed by a nonlinear deformation transformation in stereotaxic coordinates [46,47].

Individual brain structure masks were created, as previously described [13], from the porcine brain atlas template for the following structures: whole hemispheres, primary motor cortex, premotor cortex, primary somatosensory cortex, somatosensory association cortex, caudate, putamen, and globus pallidus.

White matter tractography was evaluated using MedInria software (version 3.0). Individual brain structure masks were imported and overlaid with final tractography results in MedInria’s Region of Interest (ROI) box function in the diffusion workspace. All fibers that intersected an individual brain structure ROI, such as the primary motor cortex or hippocampus, were displayed and measured. The resulting fibers were only those that intersected each grey matter brain structure and were then evaluated for ADC and FA values.

### 2.5. Gait Analysis

Before weaning, piglets were acclimated to the gait track by freely roaming along the mat in littermate groups for approximately 15 min per day for five consecutive days, then were individually acclimated to the mat for the following five days. Upon weaning, at three weeks of age, pigs were trained to travel across the gait track in a consistent, two beat trot. Individual piglet training took place for 15 min per day for five consecutive days in which consistency was reinforced with littermate social interaction at the end of the gait mat as positive reinforcement. Gait was collected at 7 DPS and piglets were approximately five weeks of age. During the collection time point, pigs traveled across the gait track until five usable trials were collected or for a maximum period of 15 min.

Individual gait trials were selected for analysis if they showed less than 10% variability in stride cycle velocity throughout the trial. Furthermore, for a trial to be included in analysis, it had to have an average stride cycle velocity that fell within 20% of the mean velocity for its respective collection day. Data were recorded using a GAITFour electronic, pressure-sensitive mat (CIR Systems, Inc., Franklin, NJ, USA) that was 7.01 m long and 0.85 m wide. The mat contains a 6.10 m × 0.61 m active area with a total of 23,040 sensors. Gait was then semi-automatically analyzed using GAITFour Software (version 4.9) to provide quantitative measurements for each limb. To account for individual pig variation, all measurements were normalized to pre-surgery values. Parameters analyzed for each limb included:

- Stride Length: Distance between successive ground contact of the same hoof.
- Step Length: Distance between corresponding successive points of contact of opposing hooves.
- Step Time: Time from initial contact of a hoof to the initial contact of the opposite hoof.

### 2.6. Statistical Analysis

All MRI and gait data were analyzed using Minitab^®^ Statistical Software (Version 21.1.1.0, State College, PA, USA). One-tailed two sample *t*-tests were used to compare treatment groups. All data are presented as mean ± standard error of the mean (SEM). *p*-values of *p* < 0.05 were considered statistically significant and *p* < 0.10 were considered trending. All data are available upon request to the corresponding author.

## 3. Results

### 3.1. T2W Sequences Detect Significant Ipsilateral Lesioning following TBI

**Figure 1:**
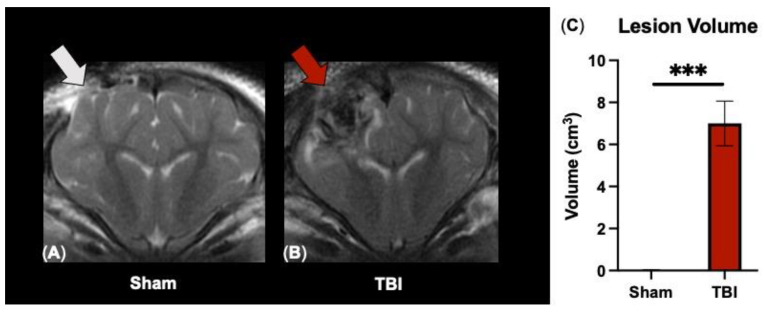
Conventional T2W MRI detects significant lesioning localized predominantly to the ipsilateral hemisphere. Representative T2W MRI images show a craniectomy site (gray arrow; (**A**)) in sham animals and a heterogenous lesion containing hypointense and hyperintense regions (red arrow; (**B**)) in TBI animals (**C**). Data are presented as mean ± SEM. *** *p* < 0.001.

To evaluate TBI severity, T2W sequences were analyzed at 7 DPS. As expected, ipsilateral (IL) lesion volumes were significant in TBI animals as compared to sham animals (6.92 ± 1.06 vs. 0.02 ± 0.02 cm^3^, respectively; *p* < 0.001 Figure 1C) post-surgery. Visually, lesioning was not detected in the contralateral (CL) hemisphere (Figure 1A,B). These data indicate substantial neural injury in the ipsilateral hemisphere only by structural T2W MRI analysis.

### 3.2. DTI Tractography Reveals Increased Diffusion in Fibers Intersecting Both Ipsilateral and Contralateral Motor Function-Related Brain Structures

**Figure 2:**
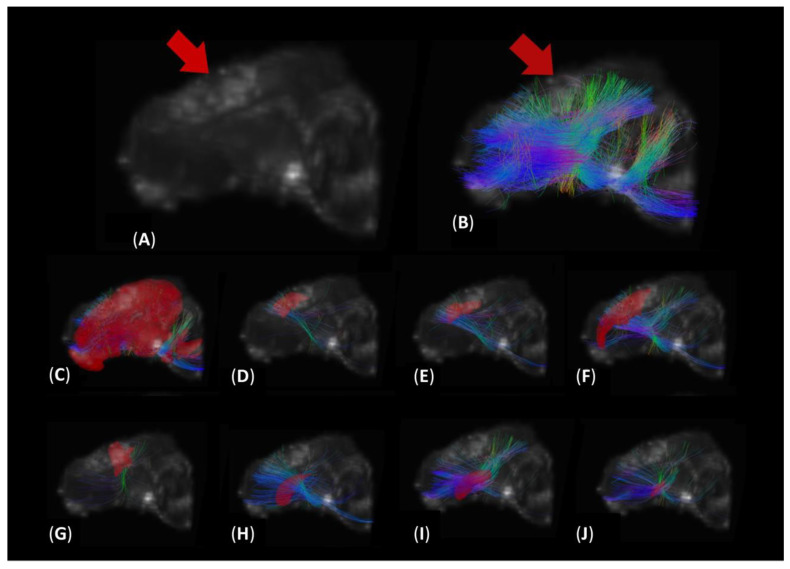
DTI tractography of white matter tracts that intersect key motor-related structures following TBI. Images depict directionality of white matter fibers of an ipsilateral sagittal view of a 3D T1W reconstruction of a representative TBI-injured piglet brain at one day post-TBI ((**A**), arrow indicates lesion at location of the primary motor cortex) with DTI tractography overlay ((**B**), arrow indicates lesion). Ipsilateral ROI (red structures) and intersecting white matter tracts were evaluated. Key brain regions involved in motor function included whole brain hemispheres (**C**), primary motor cortex (**D**), premotor cortex (**E**), primary somatosensory cortex (**F**), somatosensory association cortex (**G**), caudate (**H**), putamen (**I**), globus pallidus (**J**). Fiber tract colors are generated based on individual fiber orientation (anteroposterior fibers, green; transverse fibers, red; craniocaudal fibers, blue).

**Figure 3:**
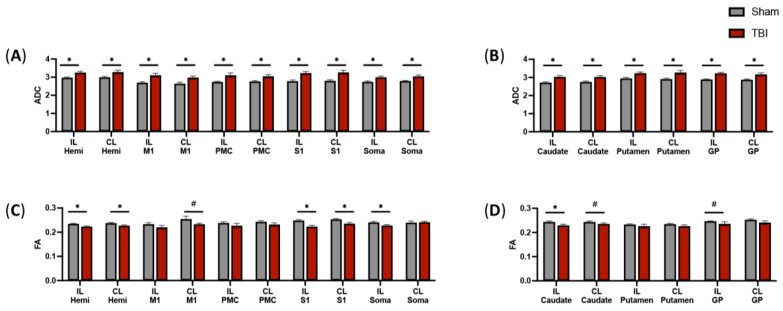
TBI leads to increased ADC and decreased FA in the white matter tracts that intersect brain regions involved in motor activity. DTI tractography was used to measure changes in ADC and FA in the intersecting white matter tracts through sensorimotor (**A**,**C**) and basal ganglia (**B**,**D**) regions involved in motor activity at acute (7 days) time points in sham and TBI-injured animals. Data are presented as mean ± SEM. * *p* < 0.05, # *p* < 0.10. CL, contralateral; GP, globus pallidus; hemi, whole brain hemispheres; IL, ipsilateral; M1, primary motor cortex; PMC, premotor cortex; S1, primary somatosensory cortex; Soma, somatosensory association cortex.

Hemispheric DTI analysis (Figure 2) revealed that the ADC values of TBI-injured animals was greater than sham animals in the fibers intersecting the IL (*p* = 0.006, Figure 3A) and CL (*p* = 0.014, Figure 3A) hemispheres. These data indicate that focal TBI localized to the ipsilateral hemisphere induced increased diffusion and white matter injury across both hemispheres, which was undetected by visual inspection of conventional T2W MRI scans.

Key sensorimotor regions measured in this study were the primary motor cortex (M1), premotor cortex (PMC), primary somatosensory cortex (S1), and somatosensory association cortex (Soma; Figure 2). TBI animals demonstrated increased ADC in the fibers intersecting the M1 (IL: *p* = 0.007, Figure 3A; CL: *p* = 0.008, Figure 3A), PMC (IL: *p* = 0.022, Figure 3A; CL: *p* = 0.020, Figure 3A), S1 (IL: *p* = 0.002, Figure 3A; CL: *p* = 0.004, Figure 3A), and soma (IL: *p* = 0.007, Figure 3A; CL: *p* = 0.005, Figure 3A) relative to sham animals.

In this study, basal ganglia structures were also evaluated for ADC measures. TBI animals demonstrated increased ADC in the fibers intersecting the caudate (IL: *p* = 0.007, Figure 3B; CL: *p* = 0.007, Figure 3B), putamen (IL: *p* = 0.007, Figure 3B; CL: *p* = 0.012, Figure 3B), and globus pallidus (IL: *p* < 0.001, Figure 3B; CL: *p* = 0.009, Figure 3B) relative to sham animals.

### 3.3. DTI Scans Indicate Decreased Fractional Anisotropy in the Intersecting Fibers of a Subset of Motor Function-Related Structures

FA is a relative measure of the directionality or degree of anisotropy of diffusion. As axons lose myelination from TBI, diffusion parallel to the fiber is decreased, therefore, FA decreases in value. Similar to changes observed in white matter tract ADC, hemispheric DTI analysis of FA showed altered values with TBI-injured animals demonstrating a lower FA relative to sham animals in fibers intersecting the IL (*p* = 0.006, Figure 3C) and CL (*p* = 0.020, Figure 3C) hemispheres. In addition, TBI animals showed a decreased FA in the intersecting fibers of the M1 (CL: *p* = 0.070, Figure 3C), S1 (IL: *p* = 0.003, Figure 3C; CL: *p* = 0.007, Figure 3C), and soma (IL: *p* = 0.020, Figure 3C) relative to sham animals. Additionally, TBI animals showed a decreased FA in the intersecting fibers of the caudate (IL: *p* = 0.020, Figure 3D; CL: *p* = 0.081, Figure 3D) and globus pallidus (CL: *p* = 0.081, Figure 3D) relative to sham animals.

### 3.4. Gait Analysis Reveals Motor Deficits in All Limbs following TBI

**Figure 4:**
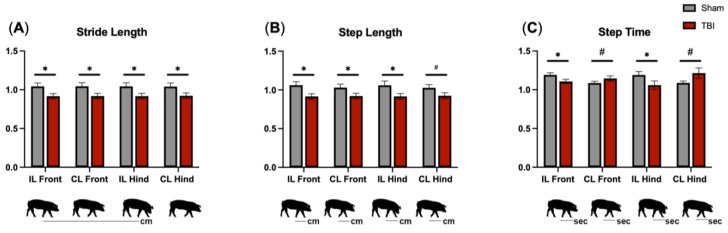
TBI leads to acute functional gait deficits. Stride length is the distance between successive ground contact of the same hoof (**A**). TBI animals exhibited an acute decrease in stride length. Step length is the distance between corresponding successive points of contact of opposing hooves (**B**). TBI animals exhibited an acute decreased step length. Step time is the time from initial contact of a hoof to the initial contact of the opposite hoof (**C**). TBI animals demonstrated a decreased step time in the ipsilateral front and hind limb as compared to sham animals. TBI animals demonstrated an increased step time as compared to sham animals in the contralateral front and hind limbs. Gait metric values post-surgery are normalized to pre-surgery metrics to account for individual variability. Data are presented as mean ± SEM. * *p* < 0.05, # *p* < 0.10.

TBI and sham piglets underwent gait data collection at 7 DPS to determine acute changes in motor function. Stride length is the distance between successive ground contact of the same hoof (Figure 4A) and often decreases following pediatric TBI [48]. TBI animals exhibited decreased stride length as compared to sham animals in all limbs (*p* < 0.05; Figure 4A). Step length measures the distance (Figure 4B) and step time measures the time (Figure 4C) between corresponding successive points of contact of opposing hooves. Step length often decreases while step time increases as TBI patients attempt to better stabilize their gait by taking slower, shorter steps while ambulating [2,48]. TBI animals showed a decrease in step length as compared to sham animals in the IL front. Furthermore, as compared to sham animals, TBI animals exhibited decreased step time in the IL front (*p* = 0.003, Figure 4C) and IL hind (*p* = 0.045, Figure 4C) limbs and a trending increased step time in the CL front (*p* = 0.089, Figure 4C) and CL hind (*p* = 0.064, Figure 4C) limbs. Together, these data demonstrate that TBI induced balance deficits and a less stable gait as evidenced by faster ipsilateral/ left limb step time as compared to the contralateral/ right limb counterpart of each diagonal pair. The step time of the less-affected ipsilateral limbs was shortened to provide more ground contact in order to improve balance and support during a gait cycle.

## 4. Discussion

In this study, we demonstrated significant changes in white matter integrity in the intersecting fibers of ipsilateral and contralateral brain regions involved in motor control in a piglet TBI model. DTI tractography of individual motor-related brain structures revealed an increase in ADC and corresponding decrease in FA that were indicative of decreased white matter integrity throughout the brain following a focal CCI TBI. These perturbations in motor region white matter tract ADC and FA were associated with significant decreases in stride and step length and increased step time, indicating a significant alteration in gait. These results support previous research that has demonstrated that altered white matter integrity in the pyramidal motor tract is associated with poor motor function [5,6,7,8,22,23,24]. In this study, it was also demonstrated that unilateral TBI resulted in bilateral white matter brain injury. This finding supported previous work in humans that showed unilateral TBI caused ipsilateral and contralateral hemisphere white matter damage due to spreading axonal degeneration [49,50]. The results of this research provide evidence that the evaluation of ADC and FA parameters in white matter fiber bundles intersecting key motor-related brain structures may serve as a powerful neuroimaging tool to predict and evaluate motor deficits in pediatric TBI.

The sensorimotor system is a complex system of neural circuitry that works in coordination to process and integrate sensory input and motor output signals with the goals of maintaining motor stability, curtailing disruptive neural feedback, and contributing to long-term changes in neural systems for motor learning [51,52]. DTI analysis has demonstrated associations between reduced white matter integrity in the pyramidal tract and balance impairments, such as reduced postural control, following pediatric TBI [5,7]. The pyramidal tract is the principal neural tract for motor function in the brain and is involved in the motor function of distal extremities [53]. This tract comprises white matter tracts from several brain regions, including the primary motor cortex and the somatosensory cortex. Immediate and constant processing of sensory and motor signals are required for proper gait coordination. Therefore, damaged fibers reduce the functionality of the sensorimotor system, ultimately manifesting as gait deficits. In agreement with these studies, our study found TBI animals exhibited decreased balance as demonstrated by step time deficits in the limbs contralateral to injury as compared to their ipsilateral counterpart of the diagonal pair. For example, the ipsilateral left front limb exhibited a shorter step time as compared to the contralateral right hind limb, indicating the need for increased ground contact for stabilization and balance in the less-affected ipsilateral limb. Therefore, the present study highlighted the likelihood of an association between gait deficits and sensorimotor signal coordination within specific brain regions in the pyramidal tract required for normal gait movement.

In this study, it was also determined that significant changes in white matter tract FA and ADC occurs in the contralateral hemisphere post-TBI, which supports findings from previous studies. A T1W reconstruction and DTI tractography overlay enabled the visualization of white matter tracts that intersect important brain regions associated with motor function, including primary motor cortex, premotor cortex, primary somatosensory cortex, somatosensory association cortex, caudate, putamen, and globus pallidus in the piglet TBI brain (Figure 2). After injury, the ipsilateral hemisphere often undergoes significant swelling that generates compressive forces on the contralateral hemisphere leading to damage (e.g., countercoup effect). Moreover, the contralateral hemisphere is exposed to inflammatory cytokines, reactive oxygen species, and other cytotoxic signaling factors generated at the primary injury site through shared cerebral spinal fluid. TBI also initiates progressive damage in which axonal degeneration spreads to the contralateral hemisphere [49,50]; therefore, both the ipsilateral and contralateral hemispheres were analyzed. Pischiutta et al. showed that severe TBI in mice induced significantly decreased FA in the contralateral corpus callosum and external capsule, which was corroborated by histological evaluation demonstrating axonal degeneration by silver staining [49]. These animals also showed significant motor deficits in beam walk testing [52]. The mechanisms associated with progressive contralateral white matter degeneration are complex. Nevertheless, it has been demonstrated that white matter degeneration in the contralateral hemisphere is, in part, influenced by anterograde degeneration [54]. In this process, damaged axons can lead to the death of the soma that is distal to the injury [54]. Future preclinical studies are warranted to further elucidate specific pathophysiological events that contribute to distal white matter degeneration and the development of treatments to limit this damage.

Potential study limitations include misalignment of white matter fibers between collected images and the atlas, which can arise from TBI pathophysiology. TBI results in lesioning, edema, and swelling that can alter the location of key brain structures being evaluated. However, our research group has previously reported the successful use of this registration pipeline in a comparable porcine ischemic stroke model to identify lesion topology information [13], which supported including a structural injury component in acute MRI assessment to improve preclinical prognostication. This current work expanded upon the developed structural MRI topology approach by incorporating a DTI fiber tract component. Another limitation of this study is the accurate differentiation of pure white matter tracts from grey matter structures, which can again be altered due to changes in structural location caused by TBI pathology, such as intracerebral swelling. However, the identification of white matter tracts used herein is based on current best practices to account for brain injury pathology in a preclinical neural injury model.

## 5. Conclusions

For the first time, this study presents evidence that acute increases in ADC and decreases in FA, likely due to reductions in white matter integrity, occurred in intersecting fibers of crucial brain regions responsible for motor control. Importantly, these white matter tract perturbations corresponded with observed motor deficits in a pediatric piglet model of TBI. These findings provide valuable data that encourage further exploration of DTI to better understand the structural changes in the brain that result in motor deficits and as potential injury and prognostic biomarkers for TBI-induced motor problems. Such investigations could potentially enable early detection of microstructural injury that may not be readily visualized on conventional structural MRI scans. This may permit more individualized rehabilitation programs to be implemented at early time points post-TBI, thus potentially improving long-term outcomes. The reported results, along with advancements in the neural injury biomarker field, contribute to the increasing prevalence and enhanced clinical utility of non-invasive biomarker imaging. Altogether, the assessment of this neuroimaging modality in a clinically relevant, large animal model may facilitate the translation of these assessments to clinical trials and strengthen the rigor of preclinical testing for novel therapeutics.

## Data Availability

All data are available upon request to the corresponding author. The data sets are not publicly available as specialized MRI programs (Osirix, MedInria, etc.) are required to open files.
